# Detection and quantification of 14 *Campylobacter *species in pet dogs reveals an increase in species richness in feces of diarrheic animals

**DOI:** 10.1186/1471-2180-10-73

**Published:** 2010-03-10

**Authors:** Bonnie Chaban, Musangu Ngeleka, Janet E Hill

**Affiliations:** 1 Department of Veterinary Microbiology, University of Saskatchewan, Saskatoon, Canada; 2 Prairie Diagnostic Services Inc, University of Saskatchewan, Saskatoon, Canada

## Abstract

**Background:**

The genus *Campylobacter *includes many species, some of which are known human and animal pathogens. Even though studies have repeatedly identified domestic dogs as a risk factor for human campylobacteriosis, our understanding of *Campylobacter *ecology in this reservoir is limited. Work to date has focused primarily on a limited number of species using culture-based methods. To expand our understanding of *Campylobacter *ecology in dogs, a collection of fecal samples from 70 healthy and 65 diarrheic pet dogs were examined for the presence and levels of 14 *Campylobacter *species using quantitative PCR.

**Results:**

It was found that 58% of healthy dogs and 97% of diarrheic dogs shed detectable levels of *Campylobacter *spp., with *C. coli, C. concisus, C. fetus, C. gracilis, C. helveticus, C. jejuni, C. lari, C. mucosalis, C. showae, C. sputorum *and *C. upsaliensis *levels significantly higher in the diarrheic population. Levels of individual *Campylobacter *species detected ranged from 10^3 ^to 10^8 ^organisms per gram of feces. In addition, many individual samples contained multiple species of *Campylobacter*, with healthy dogs carrying from 0-7 detectable species while diarrheic dogs carried from 0-12 detectable species.

**Conclusions:**

These findings represent the largest number of *Campylobacter *species specifically tested for in animals and is the first report to determine quantifiable levels of *Campylobacter *being shed from dogs. This study demonstrates that domestic dogs can carry a wide range of *Campylobacter *species naturally and that there is a notable increase in species richness detectable in the diarrheic population. With several of the detected *Campylobacter *species known or emerging pathogens, these results are relevant to both ecological and public health discussions.

## Background

*Campylobacter *is the most common bacterial cause of enteric disease worldwide [1], with an average of ten thousand Canadian and two million American cases reported annually [2,3]. Within the *Campylobacter *genus, *C. jejuni*, and its close relative *C. coli*, are reported as the most common cause of human acute bacterial enteritis. However, there is mounting evidence that other members of this genus, including *C. upsaliensis, C. concisus, C. gracilis, C. rectus *and *C. showae*, are under-appreciated for the part they play in enteritis, as well as other disease presentations [4-7]. With foodborne contamination the most recognized source for infections, ingestion of untreated water, raw milk, undercooked chicken and the cross-contamination of foods are recognized risk factors for acquiring *Campylobacter *[8-11]. In addition, many natural animal reservoirs for *Campylobacter *have been recognized, which include chicken and other poultry, wild birds, pigs, dogs, cats, sheep and cows [12]. Studies from the United States, Sweden and Australia all identify ownership of a pet dog as a risk factor for *Campylobacter *infections, especially among infants and small children [8-10]. Despite this fact, our knowledge of *Campylobacter *ecology in dogs is quite limited.

Research carried out in Europe and Asia has begun to address this question with various culture-based studies. Researchers from Taiwan, Finland, Sweden, Demark and the Netherlands have examined various dog populations and have been able to culture *C. jejuni, C. coli, C. upsaliensis, C. helveticus, C. lari *and other *Campylobacter *spp. from canine fecal samples using various growth conditions and media [13-17]. Reported carriage rates of *Campylobacter *spp. in domestic dogs ranged from 2.7% to 100% of dogs tested [13,16], with some studies reporting isolation of multiple species of *Campylobacter *from a single dog [15,17].

A major influence on our understanding of *Campylobacter *ecology in dogs has been our reliance on culture-based methods. Various selective media have been used for *Campylobacter *isolation [18], with most relying on a cocktail of antibiotics in a rich basal medium to selectively isolate *Campylobacter*. However, it has been recognized that *Campylobacter *species other than *C. coli*, *C. jejuni*, and *C. lari *are often sensitive to the antibiotics in these media [19]. Filter-based methods, in combination with nonselective media, have been shown to result in the isolation of a greater diversity of *Campylobacter *species [20], but these approaches are more labour-intensive, less selective and prone to overgrowth of fecal contaminants [19]. As our understanding of campylobacters, both pathogenic and non-pathogenic, expands beyond *C. jejuni *and *C. coli*, so must our detection methods.

The goal of this study was to take a culture-independent approach to the profiling of *Campylobacter *species in domestic pet dogs in an effort to evaluate this zoonotic reservoir and describe changes in fecal *Campylobacter *populations associated with diarrhea. Established species-specific quantitative PCR (qPCR) assays targeting the 60 kDa chaperonin (cpn60) gene of *C. coli, C. concisus, C. curvus, C. fetus, C. gracilis, C. helveticus, C. hyointestinalis, C. jejuni, C. lari, C. mucosalis, C. rectus, C. showae, C. sputorum*, and *C. upsaliensis *[21] were used to determine the *Campylobacter *profiles of 70 healthy dogs and 65 dogs with diarrhea. This study represents the largest culture-independent, quantitative investigation of *Campylobacter *in pet dogs conducted to date and is one of only a few studies to focus on North American animals.

## Results

### *Campylobacter *profiles from healthy and diarrheic dog fecal samples

Total bacterial DNA was extracted from the feces of 70 healthy dogs (from 52 households) and 65 dogs with diarrhea (from 60 households) (Additional file 1: Table S1) and tested for the presence of 14 *Campylobacter *species. Each sample was tested for an individual species in four reactions (duplicate reactions within an assay and each assay run twice). If a sample did not yield three or four detectable test values (above the assay cut-off of 10^3 ^organisms/g of feces [21]), the sample was defined as undetectable for that test. In the cases where only one or two of the four test reactions generated a detectable value, these values where at the bottom limit of assay's detection capability. Although we acknowledge that this may lead to a slight underestimation of *Campylobacter *DNA present, these samples were deemed too close to the lower assay detection limit to be confidently called as a positive sample for that test. In all other cases, positive values for a sample were within one log value of each other and all four reactions were averaged to generate the detected level of an individual *Campylobacter *species within that sample.

**Figure 1:**
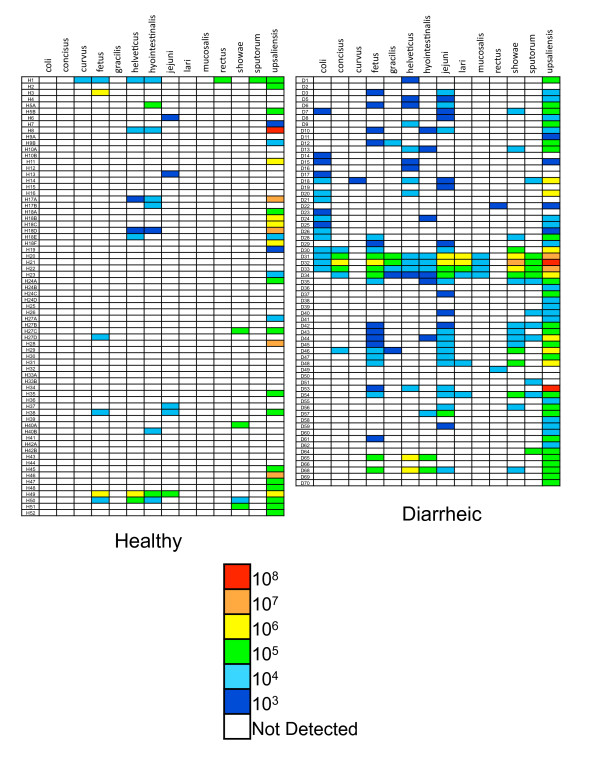
**Distribution and levels of *Campylobacter *detected in feces from healthy and diarrheic dogs**. Rows represent a single fecal sample while columns represent individual species of *Campylobacter *assayed. Coloured boxes indicate the target copies per gram of feces detected. The lower detection limit of the assays is 10^3 ^copies/g of feces [21].

Figure 1 summarizes the levels of *Campylobacter *detected in each sample for each species tested. *Campylobacter *species were detected in 56% (39/70) of healthy and 97% (63/65) of diarrheic dog feces. In a species by species comparison, significantly more diarrheic samples were positive for 11 of the 14 species assayed, with only *C. curvus, C. hyointestinalis *and *C. rectus *detection rates remaining constant between populations (Table 1). *C. upsaliensis*, commonly reported as the predominant *Campylobacter *species recovered from dogs [14-17], was also the predominant species detected in this study, with 43% (30/70) of healthy dogs and 85% (55/65) of diarrheic dogs shedding detectable levels. As well, human pathogens *C. jejuni *and *C. showae *could be detected at a low prevalence in the healthy dog population (7% (5/70) and 6% (4/70), respectively) and at a significantly higher prevalence in the diarrheic population (46% (30/65) and 28% (18/65), respectively). Also of note, *C. coli *was undetectable in the healthy dog population (0/70) but detectable in 25% (16/65) of dogs with diarrhea. Other species detected only in the diarrheic dog population were *C. concisus, C. gracilis, C. lari *and *C. mucosalis*.

**Table 1:** Numbers of healthy and diarrheic dog fecal samples positive for each species of *Campylobacter *tested.^a^

	Number of Positive samples	
	
	Healthy (/70)	Diarrheic (/65)
*C. coli*	0	16**
*C. concisus*	0	6*
*C. curvus*	1	1
*C. fetus*	6	24**
*C. gracilis*	0	6*
*C. helveticus*	7	16*
*C. hyointestinalis*	9	12
*C. jejuni*	5	30**
*C. lari*	0	6*
*C. mucosalis*	0	4*
*C. rectus*	1	2
*C. showae*	4	18**
*C. sputorum*	1	12**
*C. upsaliensis*	30	55**

^a^Statistically significant differences based on an independent t-test or Mann Whitney U test are indicated with an asterisk (p < 0.05) or double asterisk (p < 0.002).

Beyond a strictly present/absent detection of each species, the qPCR assays used in this study generate quantitative values for the number of target organisms detected per reaction [21,22]. From both the healthy and diarrheic dog populations, individual *Campylobacter *species detected in feces ranged from 10^3 ^organisms/g (the lower detection limit of the assays) to 10^8 ^organisms/g (Figure 1). Within the healthy population, only *C. fetus *and *C. upsaliensis *were detected at levels of 10^6 ^organisms/g of feces or higher. This is in contrast to the diarrheic population, where *C. concisus, C. fetus, C. helveticus, C. jejuni, C. lari, C. showae *and *C. upsaliensis *were detectable in samples at 10^6 ^organisms/g of feces or higher. Interestingly, despite the fact that more species were present at higher levels in the diarrheic population, the maximum level of any individual *Campylobacter *species detected from a sample was not more than 10^8 ^organisms/g of feces in either population (Figure 1).

**Figure 2:**
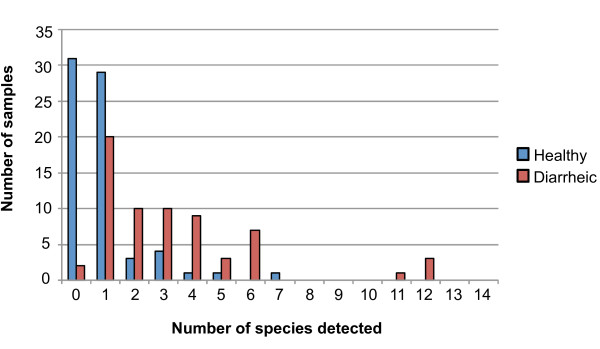
**Species richness of *Campylobacter *detected in healthy and diarrheic dog samples**.

In addition to an increase in the number of samples positive for any of the 14 *Campylobacter *species tested for, the diarrheic dog samples also had a higher species richness (Figures 1 &2). Figure 2 summarizes the number of different *Campylobacter *species detected from individual samples. For healthy dogs, 42% (31/70) of samples had no detectable *Campylobacter*, 41% (29/70) had a single species detectable and only 14% (10/70) had two or more species detectable. This compares to 3% (2/65) of diarrheic samples that had no detectable *Campylobacter*, 31% (20/65) had a single species detectable and 66% (43/65) had two or more species. Remarkably, three of the diarrheic samples tested had 12 different species of *Campylobacter *present, with individual species ranging from 10^4 ^to 10^8 ^organisms/g (Figure 1).

### Total bacteria levels in dog fecal samples

**Figure 3:**
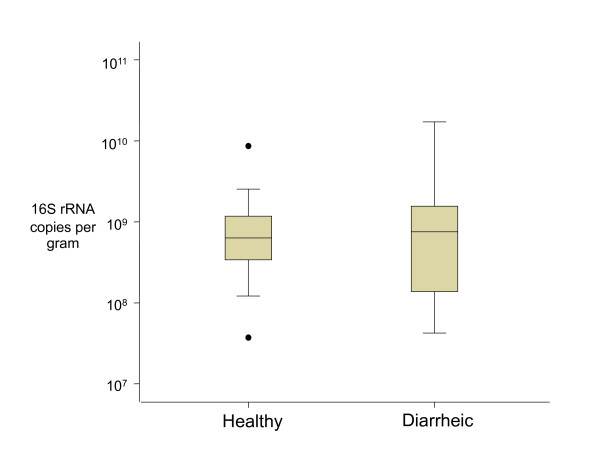
**Total bacterial 16S rRNA gene copies detected per gram of healthy and diarrheic dog feces (n = 20 for each population)**. Box plots show the 25^th ^to 75^th ^percentile range of the data within the box, with the median indicated with a line in the box. The whiskers represent the remaining quartile ranges, with outliers indicated as dots.

To determine if the difference in *Campylobacter *profiles of healthy and diarrheic dogs could be accounted for by an overall difference in fecal bacteria shedding, the total amount of detectable bacterial DNA per gram of feces was measured from each group. Twenty samples from each population were randomly selected and qPCR was performed to determine the total l6S rRNA gene copies detectable in the fecal DNA extracts. We found that both healthy and diarrheic fecal populations had approximately 10^9 ^copies/g of the 16S rRNA gene detectable (Figure 3), with no statistically significant difference between the populations (p = 0.818). This indicates that detectable bacterial levels being shed in dog feces are consistent, regardless of the animals' clinical state or the etiology of the diarrhea. Therefore, the increase in detectable *Campylobacter *shedding during diarrhea appears to be the result of an increase in the proportion of *Campylobacter *present compared to the total bacterial population.

## Discussion

*Campylobacter *species could readily be detected in feces from both the healthy and diarrheic dogs (Figure 1). From a public health perspective, several findings are of note. *C. upsaliensis*, which was the predominant species detected in this study, has been reported, second only to *C. jejuni*, as the most frequently isolated cause of campylobacteriosis in some US settings [5]. As well, many of the *Campylobacter *species examined, including known or emerging human pathogens, were detectable in both the healthy and diarrheic dog populations, with most species found at significantly higher levels in the diarrheic population (Table 1). This becomes increasingly relevant when the level of organisms detected is considered. Figure 1 highlights that in both dog populations, *Campylobacter *levels reaching 10^8 ^organisms/g of feces could be detected. With reports that the human infectious dose for campylobacteriosis by *C. jejuni *can be as low as 8 × 10^2 ^organisms ingested [23], the possibility of accidental exposure to infectious levels of *Campylobacter *from pet dogs in a household is within the realm of possibility. Taken together, our results support the findings of previous groups indicating pet dogs as a risk factor for campylobacteriosis [8-10].

From a *Campylobacter *ecology perspective, an important finding from this data is the species richness of *Campylobacter *detected, particularly in the diarrheic samples. The diarrheic dog samples examined in this study came from clinical submissions where the major clinical sign was persistent diarrhea. In the veterinary context, samples from acute cases (often caused by dietary indiscretion; i.e. eating garbage) would be submitted rarely since the diarrhea episode would resolve in a short time. The etiology of the diarrhea was not considered in our sample selection, although in many cases, intestinal bacterial overgrowth associated with increased numbers of *Clostridium perfringens *was suspected. This suggests that the apparent enrichment of *Campylobacter *populations may be related to environmental changes consistent with the physiological condition of diarrhea (which may include increased stool volume and weight, increased defecation frequency and loose stools), rather than any particular pathogen or disorder. This is consistent with reports of an increase in *C. coli *numbers in pigs suffering from swine dysentery caused by *Brachyspira hyodysenteriae*, where the reason for that *Campylobacter *increase was unclear [24]. It is possible that the healthy dogs had similar species richness, but the majority of species were present at a level below our tests' detection limits. However, the maximum levels of organisms detected were similar in the healthy and diarrheic samples (~10^8 ^organisms/g, Figure 1), suggesting that enrichment of *Campylobacter *species in the dogs with diarrhea was not uniform and that the maximum abundance of *Campylobacter *is limited in some way. Regardless of the mechanism responsible, it appears that something about the physiological state of diarrhea is favourable for *Campylobacter *species within the context of the intestinal microbiota.

## Conclusions

Pets are members of the North American family, with 37% of American and 33% of Canadian households containing pet dogs [25,26]. As our understanding of *Campylobacter *pathogenicity increases, so must our understanding of its reservoirs and ecology. Domestic dogs are recognized as a risk factor for campylobacteriosis and this report reinforces those findings. We found human pathogens like *C. jejuni, C. coli, C. upsaliensis, C. gracilis, C. concisus *and *C. showae *in dog feces, with significantly higher levels present in dogs with diarrhea. As well, we see that disturbances to the intestinal microbiota related to diarrhea have an effect on *Campylobacter *ecology. How and why this is the case, as well as how this change in *Campylobacter *distribution relates to the overall intestinal community, are areas of future investigation.

## Methods

### Sample Collection

Fecal samples from healthy dogs were submitted for analysis by pet owners from the Saskatoon, SK, Canada metropolitan area (population 250,000) (Additional file 1: Table S1). All dogs were considered healthy by their owners and had not received antibiotic therapy for at least six months prior to sample collection. Samples were collected in accordance with the University of Saskatchewan Animal Research Ethics Board (protocol #20090054). Fecal specimens from dogs suffering from diarrhea (of any etiology) were obtained from samples submitted to Prairie Diagnostic Services Inc., Saskatoon, SK for routine bacteriology and/or parasitology testing (Additional file 1: Table S1). All samples were stored at -80°C until processed for PCR analysis.

### DNA Extraction

Total bacterial DNA was extracted from fecal samples using the QIAamp DNA stool kit (Qiagen), as per manufacturer's instructions. Final DNA samples were diluted 1:10 with sterile water before analysis. This was done to improve the overall sensitivity of the assays used, which are known to be affected by PCR inhibitors carried through fecal DNA extractions [21].

### Quantitative PCR (qPCR)

The detection and quantification of the 14 species of *Campylobacter *reported was done using assays targeting the cpn60 gene using the primer sets and PCR conditions described in [21]. The lower detection limit of these assays is 10^3 ^copies/g of feces [21]. Total bacterial DNA levels were measured by quantification of the 16S rRNA gene, using the primer set SRV3-1/SRV3-2 (with an annealing temperature of 62°C) described in [27]. All assay reaction mixtures consisted of 1× iQ SYBR green supermix (Bio-Rad), 400 nmol/L concentrations of each of the appropriate primers, and 2 μL of template DNA in a final volume of 25 μL. An iCycler or MyiQ thermocycler (Bio-Rad) was used for all reactions with the following program: 95°C for 3 min, followed by 40 cycles of 15 s at 95°C, 15 s at the appropriate annealing temperature, and 15 s at 72°C. A final melt at 95°C for 1 min was done prior to a dissociation curve analysis (55°C to 95°C in 0.5°C steps for 10 s increments). Fluorescence signals were measured every cycle at the end of the annealing step and continuously during the dissociation curve analysis. The resulting data were analyzed using iQ5 optical system software (Bio-Rad). All reactions were performed in duplicate (within the assay) and each assay was performed twice, resulting in four evaluations of each sample.

### Statistical Analysis

All statistical analyses were done using SPSS software (SPSS Inc., Chicago, IL, USA). *Campylobacter *and total bacterial count data was analyzed for significance using the independent sample t-test or the Mann-Whitney U test, as appropriate.

## Authors' contributions

BC participated in sample collection, carried out all sample preparation and testing, participated in statistical analysis and drafted the manuscript. MN coordinated sample collection and participated in the design of the study and analysis. JEH conceived of the study, and participated in its design and coordination and helped to draft the manuscript. All authors read and approved the final manuscript.

## Supplementary Material

Additional file 1 **Table S1**. Additional information about the dogs from which samples were collected, including breed, age, diet and symptoms (where applicable). Relevant information about the dogs used in this study, with the healthy dog information provided by their owners at time of sample collection and the diarrheic dog information taken from case file information when sample was submitted for testing at Prairie Diagnostic Services. Click here for file
