# Supplementary material for: Detection and quantification of 14 Campylobacter species in pet dogs reveals an increase in species richness in feces of diarrheic animals
Source: BMC Microbiol. 2010 Mar 10;10:73. doi: 10.1186/1471-2180-10-73 (PMC2848019; doi:10.1186/1471-2180-10-73)
Supplement: Additional file 1 — Table S1. Additional information about the dogs from which samples were collected, including breed, age, diet and symptoms (where applicable). Relevant information about the dogs used in this study, with the healthy dog information provided by their owners at time of sample collection and the diarrheic dog information taken from case file information when sample was submitted for testing at Prairie Diagnostic Services. [file 1471-2180-10-73-S1.DOC]

**Supplementary Table 1 – Additional information about dogs from which samples were collected, including breed, age, diet and symptoms (where applicable)**

Healthy dog information provided by owners at time of sample collection. NA = Not available

| **Sample ID** | **Breed** | **Age (years)** | **Diet** | **Antibiotics last 6 months** |
| --- | --- | --- | --- | --- |
| HDS01 | Boxer | 1.3 | Commercial | no |
| HDS02 | Sheltie | 4.75 | Commercial (mostly dry), table scraps, commercial brand cookies/treats | no |
| HDS03 | Mixed | 7 | Commercial (75%), table scraps (25%) | no |
| HDS04 | Maltese x Bichon x Cockapoo | 0.5 | Commercial (premium), table scraps (healthy only - fruits/veggies/cooked chicken) | no |
| HDS05A | Mixed (lab, Norwegian elkhound), female | 1.83 | Commercial (Orijen 6 Fish, switching to Acana Pacific), table scraps (cheese, veggies, some raw meat or bones) | no |
| HDS05B | Pure Alaskan Klee Kai, male | 2.5 | Commercial (Orijen 6 Fish, switching to Acana Pacific), table scraps (cheese, veggies, some raw meat or bones) | no |
| HDS06 | Springer Spaniel | 10 | Commercial (Iams - adult maintenance) | no |
| HDS07 | Alaskan Malamute | 2 | Commercial | no |
| HDS08 | Lab/shepherd cross | 4 | Commercial (50%), table scraps (50%) | no |
| HDS09A | Lab cross | 13 | Commercial (MediCal vegetarian), table scraps (some) | no |
| HDS09B | Portuguese water dog | 0.92 | Commercial (Ripples), table scraps (some) | no |
| HDS10A | Retriever cross | 10 | Commercial (Hills - Senior diet), dog biscuits | no |
| HDS10B | Sheltie-Beagle cross | 9.5 | Commercial (Hills - Senior diet), dog biscuits | no |
| HDS11 | Yorkshire terrier x Bichon/maltese | 6 | Commercial (Caesar wet, Horizon dry), table scraps (some) | no |
| HDS12 | Dalmatian | 12.75 | Commercial (Royal Canin Skin Support) | no |
| HDS13 | Shintzu x Pekingese | 13 | Commercial (MediCal Hypoallergenic kibble) | no |
| HDS14 | Jack Russell | 3 | Commercial (Lamb & rice) | no |
| HDS15 | Portuguese water dog | 6 | Commercial (Nutros kibble) | no |
| HDS16 | Cockapoo | 4.5 | Commercial | no |
| HDS17A | Labrador retriever | 4.5 | Raw food diet (venison, beef, chicken, fruits & vegetables), Commercial dog treats, deer droppings, and generally anything interesting (grass, dirt, compost, etc) | no |
| HDS17B | Labrador retriever | 4.5 | Commercial (Iams weight control proactive health, treats), vegetables | no |
| HDS18A | Labrador retriever | 2 | Raw food diet (venison, beef, chicken, fruits & vegetables), Commercial dog treats, deer droppings, and generally anything interesting (grass, dirt, compost, etc) | no |
| HDS18B | Labrador retriever | 4.5 | Raw food diet (venison, beef, chicken, fruits & vegetables), Commercial dog treats, deer droppings, and generally anything interesting (grass, dirt, compost, etc) | no |
| HDS18C | Labrador retriever | 8.5 | Raw food diet (venison, beef, chicken, fruits & vegetables), Commercial dog treats, deer droppings, and generally anything interesting (grass, dirt, compost, etc) | no |
| HDS18D | Labrador retriever | 13.5 | Raw food diet (venison, beef, chicken, fruits & vegetables), Commercial dog treats, deer droppings, and generally anything interesting (grass, dirt, compost, etc) | no |
| HDS18E | Labrador retriever | 8.5 | Raw food diet (venison, beef, chicken, fruits & vegetables), Commerical dog treats, deer droppings, and generally anything interesting (grass, dirt, compost, etc) | no |
| HDS18F | Beagle | 5.5 | Raw food diet (venison, beef, chicken, fruits & vegetables), Commercial dog treats, deer droppings, and generally anything interesting (grass, dirt, compost, etc) | no |
| HDS19 | Chesapeake Bay Retriever | 1.75 | Commercial | no |
| HDS20 | Bichon | 14 | Commercial | no |
| HDS21 | Cocker spaniel | 2.5 | Commercial | no |
| HDS22 | Lab/border collie | 13 | Commercial (Purina) | no |
| HDS23 | Pitbull cross | 0.17 | Commercial (Medical development) | no |
| HDS24A | Shetland sheepdog | 2.5 | Commercial | no |
| HDS24B | Shetland sheepdog | 8 | Commercial | no |
| HDS24C | Shetland sheepdog | 6 | Commercial | no |
| HDS24D | Shetland sheepdog | 12 | Commercial (Iams low residue) | no |
| HDS25 | Husky | 0.42 | Commercial | no |
| HDS26 | Labradoodle | 2.1 | Raw food diet | yes (?) |
| HDS27A | American eskimo x shetland sheepdog x possible border collie | 9 | Commercial (Horizon, Authority, Nutro, Iams), Raw food (beef, chick, pork, rarely fish), vegetables, table scraps, added vitamin supplements | no |
| HDS27B | Shetland sheepdog | 1.83 | Commercial (Horizon, Authority, Nutro, Iams), Raw food (beef, chick, pork, rarely fish), vegetables, table scraps, added vitamin supplements | no |
| HDS27C | Border collie x springer spaniel | 1 | Commercial (Horizon, Authority, Nutro, Iams), Raw food (beef, chick, pork, rarely fish), vegetables, table scraps, added vitamin supplements | no |
| HDS27D | Shetland sheepdog | 8.5 | Commercial (Horizon, Authority, Nutro, Iams), Raw food (beef, chick, pork, rarely fish), vegetables, table scraps, added vitamin supplements | no |
| HDS28 | Besoir; pappilli - poodle cross | 7 | Commercial, table scraps (chili, wieners) | no |
| HDS29 | Pure German shepherd | 8 | Commercial (Pedigree), table scraps | no |
| HDS30 | Border collie x sled dog | 3 | Commercial (Evo kibble), table scraps (cooked beef/tuna/barley/rice/vegetables) | no |
| HDS31 | Vizsla | 9 | Commercial (Iams), table scraps | no |
| HDS32 | Golden Retriever | 12 | Commercial | no |
| HDS33A | Mini schnauzer | 7 | Commercial (Horizon - Rosthern Topend, Cosco duck jerky) | no |
| HDS33B | Mini schnauzer | 8 | Commercial (Horizon - Rosthern Topend, Cosco duck jerky) | no |
| HDS34 | Cockapoo | 3.25 | Commercial (Innova) | no |
| HDS35 | Chocolate lab | 7 | Commercial | no |
| HDS36 | Australian blue heeler (Australian cattle dog) | 6 | Commercial, table scraps (meat) | yes - (ear lobe infection) |
| HDS37 | Labrador | 3 | Commercial | no |
| HDS38 | Labrador | 4 | Commercial (Medical gastro) | no |
| HDS39 | Mini schnauzer | 8 | Commercial | no |
| HDS40A | Port water dog | 7 | Commercial | NA |
| HDS40B | Port water dog | 5 | Commercial | NA |
| HDS41 | Boxer | 0.5 | Commercial (Eukanuba Large Breed Puppy) | no |
| HDS42A | Domberman cross | 6 | Commercial (PC Nutrition 1st) | no |
| HDS42B | Belgian Malinois cross | 6 | Commercial (PC Nutrition 1st) | no |
| HDS43 | Rottweiler x shepherd | 1.75 | Commercial, Raw food, table scraps | no |
| HDS44 | Alaskan Malamute | 4 | Commercial (Science Diet), table scraps (peanut butter, apples), milk bone | no |
| HDS45 | Coonhound | 1.33 | Commercial (Innova large breed), tripett | no |
| HDS46 | Shepherd cross | 3.5 | Commercial (Iams & Purina) | no |
| HDS47 | Maltese/apso lapso | 5.5 | Commercial, homemade treats | no |
| HDS48 | Mini schnauzer | 5 | Commercial | yes - 4 months previous |
| HDS49 | Bichon/shihtzo | 0.67 | Commercial | no |
| HDS50 | Shih Tzu | NA | Commercial (Medical R/C) | no |
| HDS51 | Shih Tzu cross | 5 | Commercial | no |
| HDS52 | Shih Tzu | 5 | Commercial | no |

Diarrheic dog information was taken from case file information when sample was submitted for testing at Prairie Diagnostic Services. NA = Not available.

| **Sample ID** | **Breed** | **Age (years)** | **Notes** |
| --- | --- | --- | --- |
| DDS01 | Labrador retriever | 8 | loose stool |
| DDS02 | German shepherd | 7 |  |
| DDS03 | Siberian husky | 0.33 | loose stool |
| DDS05 | bichon frise cross | 12 |  |
| DDS06 | Terrier | 0.15 | bloody stool |
| DDS07 | Boxer | 7 | watery stool |
| DDS08 | Golden retriever | 0.67 |  |
| DDS09 | English cocker spaniel | 0.21 | soft stool |
| DDS10 | Labrador retriever cross | 0.25 |  |
| DDS11 | Cocker spaniel cross | 9 |  |
| DDS12 | German shepherd | NA | 12-G; same household as DDS13 and DDS14 |
| DDS13 | German shepherd | NA | 13-D; same household as DDS12 and DDS14 |
| DDS14 | German shepherd | NA | 14-R; same household as DDS12 and DDS13 |
| DDS15 | NA | 2 | diarrhea resolved |
| DDS16 | Miniature schnauzer | 6 | bloody stool |
| DDS17 | Basenji | 2 | soft stool with mucous |
| DDS18 | Labrador retriever | 2 | 18-B; same household as DDS19 |
| DDS19 | Labrador retriever | 2 | 19-W; same household as DDS18 |
| DDS20 | Toy poodle cross | 0.67 |  |
| DDS21 | Doberman Pinscher cross | 1 |  |
| DDS22 | bichon frise cross | 7 |  |
| DDS23 | Labrador retriever | 0.58 |  |
| DDS24 | NA | NA | 24-K; loose stool; same household as DDS25 |
| DDS25 | NA | NA | 25-M; loose stool; same household as DDS24 |
| DDS26 | American Eskimo (spitz) | 0.33 | soft, loose stool |
| DDS28 | Shih Tzu cross | 0.67 | no blood or mucous in stool |
| DDS29 | English setter | 8 | very liquid stool |
| DDS30 | Alaskan Malamute | 0.15 | very loose stool |
| DDS31 | Boxer cross | 0.23 | soft stool |
| DDS32 | Golden Retriever | 0.25 |  |
| DDS33 | NA | NA | stool with and without blood |
| DDS34 | Siberian husky | 0.58 |  |
| DDS35 | Australian shepherd | 1 |  |
| DDS36 | Australian heeler | 3 | little blood in stool |
| DDS37 | Dachshund | 8 | bloody stool |
| DDS38 | Great Pyrenees | 0.75 | runny stool; same household as DDS39 |
| DDS39 | Great Pyrenees | 0.75 | same household as DDS38 |
| DDS40 | Shih Tzu cross | 5 | bloody stool |
| DDS41 | German shepherd | 4 |  |
| DDS42 | Shih Tzu | 1 |  |
| DDS43 | German shepherd cross | 3 |  |
| DDS44 | Siberian husky | 0.75 |  |
| DDS45 | Boxer | 0.29 |  |
| DDS46 | Great Dane | 0.67 |  |
| DDS47 | Miniature Dachshund | 0.4 |  |
| DDS48 | Saint Bernard | 5 |  |
| DDS49 | Pomeranian | 1.7 |  |
| DDS50 | Maltese cross | 0.33 | soft stool |
| DDS51 | Pomeranian | 1.75 | loose stool |
| DDS53 | Rottweiler cross | 2 | bloody stool |
| DDS54 | Labrador retriever | 0.12 | bloody stool |
| DDS55 | German shepherd | 0.5 | loose stool with mucous +/- blood |
| DDS56 | Border collie cross | 2 |  |
| DDS57 | Golden retriever | 0.4 |  |
| DDS58 | Boston Terrier | NA | tested for shipping |
| DDS59 | Samoyed | 13 | bloody stool |
| DDS60 | Cocker spaniel cross | 0.31 | mucous and blood in stool |
| DDS61 | NA | NA | NA |
| DDS62 | NA | NA | NA |
| DDS64 | American Eskimo (spitz) | 0.75 |  |
| DDS65 | Siberian husky cross | NA | new sled dog |
| DDS66 | NA | NA |  |
| DDS68 | Labrador retriever cross | NA |  |
| DDS69 | Boston Terrier | NA | bloody stool |
| DDS70 | Collie cross | NA |  |
